# Understanding Weight Loss via Online Discussions: Content Analysis of Reddit Posts Using Topic Modeling and Word Clustering Techniques

**DOI:** 10.2196/13745

**Published:** 2020-06-08

**Authors:** Yang Liu, Zhijun Yin

**Affiliations:** 1 College of Computer Science and Technology Changchun Normal University Changchun China; 2 Department of Biomedical Informatics Vanderbilt University Medical Center Nashville, TN United States; 3 Department of Electrical Engineering and Computer Science Vanderbilt University Nashville, TN United States

**Keywords:** weight loss, online health community, machine learning, topic modeling, word2vec, hierarchical clustering, consumer health

## Abstract

**Background:**

Maintaining a healthy weight can reduce the risk of developing many diseases, including type 2 diabetes, hypertension, and certain types of cancers. Online social media platforms are popular among people seeking social support regarding weight loss and sharing their weight loss experiences, which provides opportunities for learning about weight loss behaviors.

**Objective:**

This study aimed to investigate the extent to which the content posted by users in the *r/loseit* subreddit, an online community for discussing weight loss, and online interactions were associated with their weight loss in terms of the number of replies and votes that these users received.

**Methods:**

All posts that were published before January 2018 in r/loseit were collected. We focused on users who revealed their start weight, current weight, and goal weight and were active in this online community for at least 30 days. A topic modeling technique and a hierarchical clustering algorithm were used to obtain both global topics and local word semantic clusters. Finally, we used a regression model to learn the association between weight loss and topics, word semantic clusters, and online interactions.

**Results:**

Our data comprised 477,904 posts that were published by 7660 users within a span of 7 years. We identified 25 topics, including food and drinks, calories, exercises, family members and friends, and communication. Our results showed that the start weight (β=.823; *P*<.001), active days (β=.017; *P*=.009), and median number of votes (β=.263; *P*=.02), mentions of exercises (β=.145; *P*<.001), and nutrition (β=.120; *P*<.001) were associated with higher weight loss. Users who lost more weight might be motivated by the negative emotions (β=−.098; *P*<.001) that they experienced before starting the journey of weight loss. In contrast, users who mentioned vacations (β=−.108; *P*=.005) and payments (β=−.112; *P*=.001) tended to experience relatively less weight loss. Mentions of family members (β=−.031; *P*=.03) and employment status (β=−.041; *P*=.03) were associated with less weight loss as well.

**Conclusions:**

Our study showed that both online interactions and offline activities were associated with weight loss, suggesting that future interventions based on existing online platforms should focus on both aspects. Our findings suggest that online personal health data can be used to learn about health-related behaviors effectively.

## Introduction

### Background

Maintaining a healthy weight can reduce the risk of developing many diseases, including type 2 diabetes, hypertension, heart disease and strokes, kidney diseases, and certain types of cancers [1-6]. Unfortunately, overweight or obesity has nowadays become a public health crisis that impacts many Americans. For example, it was reported that nearly 94 million US adults were affected by obesity in 2015 and the annual medical cost was approximately US $150 billion [7]. To promote public health and help control overweight and obesity, it is critical to understand what factors are associated with weight loss and design effective weight loss interventions.

Over the past decade, people have been increasingly leveraging online social media platforms to share personal experiences and seek social support regarding weight loss, understand the impact of obesity, and learn their interpretation as contributing factors to a healthy life [8]. This huge amount of online information enables health care providers and researchers to gain insights into both public and personal health. For example, studies showed that what people shared on Instagram and Twitter could be used to effectively assess obesity prevalence in the United States [9,10]. While a content analysis showed that Twitter users were more likely to discuss weight loss during and after holidays [11], a survey-based study suggested that the interactions on Twitter were too brief and shallow [12], which might constrain its users to gain deep social support. This is, however, a major motivation for people to engage in other online weight loss forums or communities [13].

As such, recent studies, which aimed to prevent obesity and promote healthy weight loss, tended to incorporate online social media platforms into their intervention design, but the effects were found to be mixed. While some investigations showed that these online platforms had the potential for an innovative weight loss intervention [14,15], others found their effects were limited because of a low retention and engagement rate [16,17]. Moreover, a meta-analysis of over 2000 studies concluded that the effects of the interventions incorporating online social networks were very modest in improving health-promoting behaviors [18]. This suggested that before designing interventions based on qualitative evidence that online support and engagement are helpful [19], a quantitative analysis is necessary in determining how online interactions with other users (as a potential external influential factor), and a user’s offline activities recorded in online discussions (as a potential internal driving factor), are associated with their weight loss.

### Current Research and Its Limitation

It should be noted that there were studies showing that consistent online activities (eg, updating progress in weight loss and interacting with others) in online weight loss programs or training were associated with higher weight loss [20-22]. Particularly, a study using data from *r/loseit* showed that higher BMI levels and higher online activities were associated with more weight loss [23]. Similarly, another study based on causal inference found that, on average, users who received comments in *r/loseit* lost 9 lb more than users who did not receive any comments [24]. Although online interactions were shown to have a significant impact on weight loss [25], existing studies focused less on the content of posts. While both the aforementioned studies used topic modeling to extract topics, they merely focused on the most popular ones. This method, however, was too general to identify detailed offline activities that were disclosed in such a large number of posts. Moreover, these studies used the data that were generated during 2010 and 2014, which, as we showed later, consisted of only a small fraction of the posts published in *r/loseit*.

In fact, highlighting both online interactions and personal offline activities aligns with social cognitive theory (SCT) [26]. The theory emphasizes that external and internal social reinforcement together lead to behavior change in a dynamic fashion and is often applied to guild the design of effective intervention strategies [27]. This suggests that focusing on either online interactions or personal offline activities but not both might lead to an incomplete view of the roles of online communities in the process of weight loss, but this needs to be examined and confirmed with evidence.

### Objective

Therefore, considering the limitation of previous studies and inspired by SCT, we aimed to investigate the extent to which the offline activities communicated by users in the *r/loseit* subreddit, an online community for discussing weight loss, and online interactions were associated with their weight loss. Specially, we focused on a data set consisting of 477,904 posts that were published by 7660 users before January 2018 in *r/loseit*. We used the self-reported weight change to measure weight loss and the average number of comments and votes that they received from other users to characterize online interactions. We applied both topic modeling and word clustering to obtain detailed and interpretable contributing factors from online posts. Finally, we used a linear regression model to quantitively examine the association between online interactions, factors described in online discussions, and the amount of weight loss.

Our work provided evidence that an online social media platform can serve as an effective data source to understand weight loss, and our findings implied that in future weight loss analyses or interventions, online interactions should be considered as a factor that influences long-term self-efficacy.

## Methods

### Data

Our data were collected from *r/loseit*, a subreddit focusing on weight loss in Reddit, an online discussion platform. Within the subreddit, users can either publish a *submission* to start a new discussion thread or make comments on either a submission or another comment to an existing discussion thread. For simplicity, we used the word *post* to denote either a submission or a comment when we did not differentiate them. In addition, Reddit users can upvote or downvote a comment but can only upvote a submission. Furthermore, users in many subreddits are allowed to enter text or symbols into a *flare*, which appears next to their usernames in a post, to show some basic information of themselves. For example, in *r/loseit*, users can show their *start weight*, *current weight*, and *goal weight* and even their *gender*, *age,* and *height* information in flairs. However, as creating a *flare* is not required, users can ignore it when publishing a post.

In this study, we used the Python Reddit API Wrapper python package (version 5.3.0) to extract data from the Reddit application programming interface. Specifically, we collected all the posts in *r/loseit* that were published before January 13, 2018. We used the flares tagged with usernames to confine our study cohorts to those users who disclosed their start, current, and goal weights and were active for at least 30 days in this subreddit [23]. It should be noted that we did not ask for permission to use the data from the Reddit community because the data are publicly accessible. However, we never tried to identify any Reddit user by linking their Reddit data with additional data sets. All the results, and post samples presented in this paper, were carefully examined and revised such that no personally identifiable information was disclosed.

### Topic Modeling and Word Semantic Clustering

Owing to high dimensionality, noise, and ambiguity of natural language text, processing and analyzing raw post content are often challenging, and the analyzed results are difficult to interpret. In this study, we used 2 types of methods to mitigate this problem: topic modeling and word semantic clustering based on low dimensional representation (eg, word2vec) [28]. While topic modeling can help identify themes in a global context, word semantic clusters can provide more detailed concepts in a local context [29].

Specifically, we used the implementation of latent Dirichlet allocation (LDA) in Mallet (version 2.0.8) to identify the main topics of online discussions in *r/loseit* [30]. Since LDA is an unsupervised algorithm, we relied on a coherence score to determine the optimal number of topics [31]. In this study, we trained LDA models for 5 to 75 topics (with a step size of 5) on all of the posts and chose the number of topics that was corresponding to the highest coherence score. To mitigate word sparsity, we only kept nouns, verbs, adjectives, and adverbs.

To obtain word semantic clusters, we relied on the Google pretrained word2vec model because our data set was not large enough to train an accurate word2vec model. We relied on the standard deviation of cluster size to determine the optimal number of clusters [29]. Specifically, we used a hierarchical clustering algorithm with 25 to 1000 clusters (with a step size of 25) and used the elbow rule to the standard deviation of the number of words in clusters. Intuitively, a large word cluster is more likely to contain multiple concepts, while a small word cluster is more likely to have little contribution to reducing hundreds of thousands of word dimensions [29].

### Regression Analysis

In this study, we investigated the association between weight loss and online discussions by using a linear regression model. Specifically, we characterized a user’s online discussion by using the following predictors:

- The days that the user was active in the subreddit.
- The number of posts that the user published.
- The topics conveyed in the posts, measured by topic distribution.
- The word semantic clusters, measured by term frequency–inverse document frequency values.
- The median karma score or votes that the user received for each post, measured by subtracting the number of upvotes from the number of downvotes [32].
- The median number of comments for each post that the user published.

We used weight loss, measured by subtracting the start weight from the current weight, as the outcome variable of the regression model. As the distribution of the weight loss variable is right-skewed, we log-transformed it before feeding it into the regression model. All the predictors were normalized and scaled into a range of (0, 1). It should be noted that the active days and the number of comments were also log-transformed because of their right-skewed distributions.

Considering that a person who had higher weight at the beginning is more likely to lose more weight, we also introduced *start weight* as a control variable in the model. Before applying the regression analysis, we used the *findCorrelation* function, as implemented in the caret R package (version 6.0-81), with a cutoff of 0.3 to remove correlated predictors. We reported predictors with a statistical significance level of .05.

## Results

### Data Statistics

We collected 2,526,277 posts published by 205,722 users during the period between July 30, 2010, and January 13, 2018. Focusing on the users who disclosed their start, current, and goal weights in flairs, we finally obtained 7660 users with a total of 477,904 posts. These posts included 16,332 submissions and 461,572 comments. Table 1 summarizes the basic statistics of key factors regarding this study cohort. From the table, we observed that most posts received a small number of comments and karma scores.

**Figure 1:**
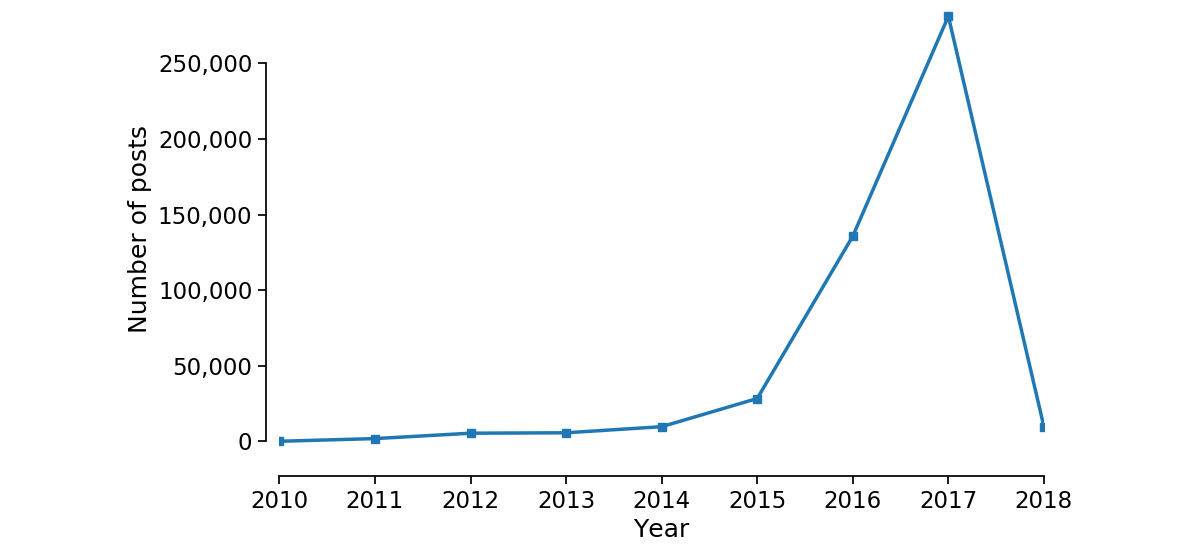
The number of posts published in this subreddit in each year (from July 30, 2010, to January 13, 2018).

Figure 1 shows the number of posts for each year during 2010 and 2018. From the figure, we can see that the number of posts surged after 2014. It should be noted that the number of posts in 2018 was small because there were only 2-week data before we stopped data collection.

**Table 1:** Basic statistics of the study cohort.

Statistics	25th percentile	Median	75th percentile
The number of posts per user, n	8	19	52
The number of words per post, n	18	39	78
The start weight (lb)	183.0	220.0	265.0
Weight loss (lb)	16.0	30.0	52.0
Active days, n	21	89	281
The number of comments for each post, n	1	1	1
The karma score for each post, n	1	2	3

### Topics Discovered in Online Discussions

We identified 25 topics that were corresponding to the highest coherence score (Multimedia Appendix 1). Table 2 shows the inferred topics, their marginal distribution, and the most relevant terms. The marginal distribution of a topic was measured by the probability that the topic was sampled from online discussions, while the relevance of a term was measured by the probability that it was sampled from a topic. The identifier of each topic was named based on the descending order of their topic distribution. For example, topic T1, talking about drinks, had the highest distribution, while topic T25, one of the weight change–related topics, had the lowest topic distribution.

We also manually summarized the 25 topics into 11 categories and provided the associated labels in Table 2. The table shows that people in this subreddit often talked about food and drinks, exercise, calorie, clothes, time, health issues, weight change, feelings, plans, and communication.

**Table 2:** The 25 topics that were identified from r/loseit. The sample terms were ordered based on their relevance to the topic. The distribution of each topic is calculated on all the posts published by these users.

Label and ID		Most relevant terms	Distribution (probability)
**Food or drinks**			
	T2	water, drink, lot, drinking, make, soda, cut, diet, beer, bit, thing, alcohol	.053
	T11	eat, food, meal, eating, lunch, dinner, hungry, calorie, breakfast, pizza	.051
	T21	food, diet, eating, eat, low, protein, fat, calorie, lot, high, carbs, cico	.049
	T22	veggie, chicken, salad, cheese, add, recipe, make, cook, egg, rice, meat	.048
**Health issues**			
	T5	doctor, body, issue, problem, health, level, pain, energy, effect, surgery	.052
**Family or friends**			
	T6	friend, family, guy, kid, mom, school, husband, life, live, told, girl	.052
**Calorie**			
	T7	calorie, deficit, exercise, tdee, eat, counting, count, eating, daily, mfp	.052
	T8	calorie, chocolate, sugar, sweet, snack, coffee, craving, bar, fruit, cal, cup	.051
**Exercise**			
	T9	run, running, walk, minute, time, walking, mile, step, hour, half, long	.051
	T16	gym, exercise, muscle, workout, start, working, work, body, cardio	.049
**Weight change**			
	T3	lb, year, lost, month, started, pound, back, ago, gained, weight, starting	.052
	T17	weight, lose, loss, fat, body, gain, healthy, bmi, height, normal, range	.049
	T25	week, scale, weight, pound, number, plateau, drop, weighing, daily	.043
**Feelings**			
	T12	thing, work, make, find, lot, time, hard, put, give, won, easy, worry	.051
	T13	goal, hit, progress, end, picture, feel, close, happy, set, time, challenge	.050
	T14	feel, feeling, eating, binge, bad, stop, time, control, hard, stress, struggle	.050
**Communication or encouragement**			
	T1	good, pretty, yeah, luck, lol, idea, feel, kind, sound, guess, bit, haha, stuff	.053
	T10	people, person, health, comment, understand, talk, mental, life, care	.051
	T23	post, read, check, https_www, question, loseit, app, mfp, reddit, http	.048
	T24	great, love, awesome, job, amazing, congrats, hope, sound, glad, similar	.046
**Plan or decision**			
	T4	day, week, back, track, maintenance, couple, plan, log, logging, cheat	.052
	T18	change, time, make, start, long, life, habit, making, thing, choice, healthy	.049
	T20	time, thought, made, felt, wanted, started, back, needed, decided, found	.049
**Clothes**			
	T19	fit, size, clothes, big, wear, buy, pant, bought, short, shirt, dress, store	.049
**Time**			
	T15	today, morning, night, yesterday, work, tomorrow, weekend, day, home	.050

### Regression Analysis

**Figure 2:**
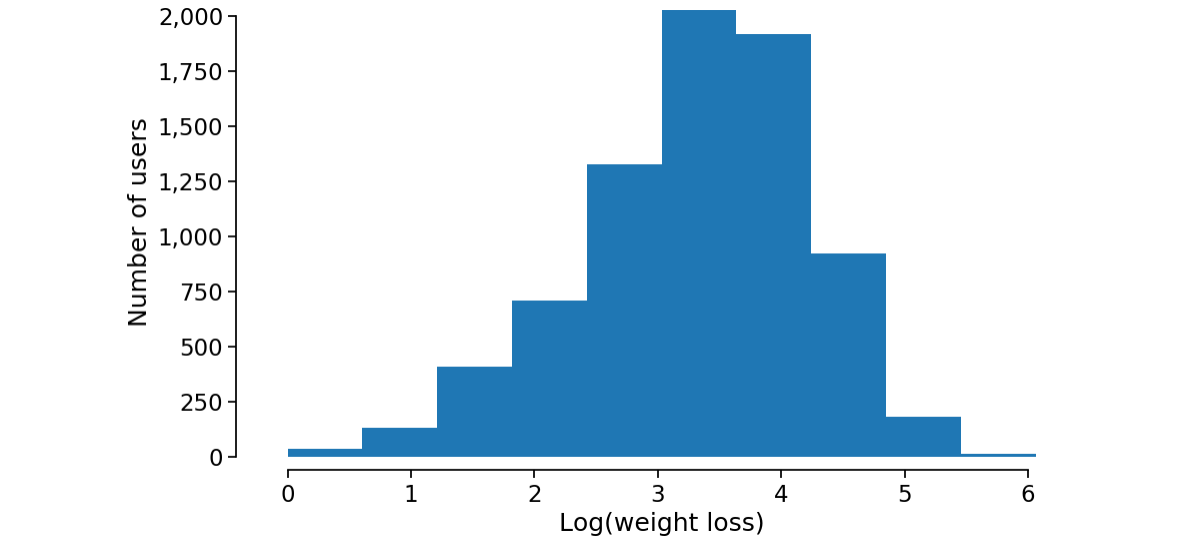
Histogram of log-transformed weight loss.

We chose 425 as the optimal number of word clusters based on the elbow rule (Multimedia Appendix 1). The word semantic clusters, together with other proposed predictors, were applied to fitting a linear regression model. After examining the feature correlation, we included 6 topics and 402 word semantic clusters into the regression model. Figure 2 shows the distribution of the log-transformed weight loss, which matches the loose constraint of applying a linear regression model.

#### Goodness of Fit and Non–content Predictors

The fitted linear regression model had an adjusted *R^2^*=0.315, *F*_7,191_=9.553, and *P*<.001, suggesting that the model with proposed predictors predicted weight loss better than the basic intercept-only model. Among the non–content-related predictors, the *start weight* (β=.823; *P*<.001), the *active days* in the subreddit (β=.02; *P*=.009), and the *median karma score* (β=.263; *P*=.02) were associated with higher weight loss. However, the *median number of comments* was not significantly associated with weight loss in our analysis (β=.001; *P*=.95).

#### Topic-Related Predictors

There were 3 topics that were found to be significantly associated with weight loss. Topics T16 (exercise in the gym) and T19 (purchase of clothes) were associated with higher weight loss (β=.072; *P*=.007 and β=.080; *P*<.001, respectively), while topic T7 (counting calorie) was associated with lower weight loss (β=−.074; *P*=.007).

#### Word Semantic Cluster–Related Predictors

Table 3 shows word semantic clusters that were significantly associated with higher weight loss, which are summarized below.

**Table 3:** Word semantic clusters that were statistically significant with respect to weight loss. Sample words are presented based on their distances to the center of a cluster in ascending order.

ID	Sample words	Beta	z score	SD	*P* value
C258	jacket, sweater, socks, sweaters, shirt, shirts, scarf, plaid, sweatshirts, jackets, coats, sandals, sweatshirt	.145	6.126	0.024	<.001
C383	emulate, replicate, magic, magical, superhuman, miraculous, recreate, miracle, feats, imitate, miracles	.130	4.699	0.028	<.001
C46	zelda, kinect, newb, wii, noob, mod, addons, nintendo, mmo, mario, xbox, mods, gamers, sims, addon, gamer	.129	1.988	0.065	.047
C93	cardio, aerobic, yoga, boxercise, gymming, pilates, plyometrics, treadmill, stairmaster, calisthenics	.128	4.567	0.028	<.001
C301	protein, metabolism, metabolic, leptin, lipids, proteins, serotonin, enzyme, enzymes, hormone, ghrelin	.120	5.143	0.023	<.001
C334	angst, frustration, anguish, anxiety, unhappiness, despair, bitterness, anger, loathing, resentment	.098	3.826	0.025	<.001
C304	musical, orchestra, opera, rehearsal, rehearsals, singers, performances, theater, choir, audition, artistic	.093	3.163	0.029	.002
C203	coach, powerlifting, teammates, captain, coached, squad, powerlifter, weightlifting, players, coaches	.085	2.027	0.042	.04
C110	younger, age, youngest, older, aged, ages, novice, old, beginners, beginner, oldest, lifelong, intermediate	.081	4.643	0.017	<.001
C24	straightforward, efficient, economical, adaptable, intuitive, inexpensive, sophisticated, expensive, easy	.081	4.405	0.018	<.001
C143	copper, zinc, moly, gold, ore, metals, tungsten, mineral, nickel, minerals, lithium, silver, metal, mines	.073	2.860	0.025	.004
C193	flower, flowers, bulb, dandelion, fragrant, roses, twigs, leaf, daffodils, buds, hibiscus, tree, crocus, trees, moss	.073	2.520	0.029	.01
C291	restaurant, restaurants, diner, deli, resturant, café, diners, bistro, cafe, pizzeria, steakhouse, resturants	.065	2.153	0.03	.03
C368	fitted, styled, proportioned, equipped, smooth, positioned, engineered, designed, smoother, molded	.059	2.584	0.023	.01
C293	discarded, trash, garbage, trashcan, bins, dumpster, recycle, dump, rubbish, scraps, leftovers, recycled, bin	.057	2.243	0.025	.03
C108	socialize, invite, catered, inviting, socializing, catering, cater, hosting, caters, hosted, invited, accommodating	.055	2.982	0.018	.003
C233	driveway, pavement, sidewalk, sidewalks, road, roads, highway, street, freeway, streets, alley, intersection	.054	2.062	0.026	.04
C66	cookouts, picnics, barbecues, cookout, feasts, picnic, potluck, togethers, barbeque, barbecue, potlucks, feast	.052	2.280	0.023	.02
C285	gerd, brussel, havn, ddp, utrecht, stephan, scandinavia, bulgarian, cico, sicilian	.045	2.797	0.016	.005
C25	gluttony, indulgence, vices, gluttonous, sinful, stupidity, hedonistic, sin, decadent, indulgent, excesses, sloth	.044	2.563	0.017	.01
C200	walkable, downtown, waterfront, neighborhoods, neighborhood, park, city, touristy, parks, suburbs	.041	1.967	0.021	.049

#### Exercise-Related Clusters

Exercise-related clusters included C258 (clothes), C46 (Xbox games), C93 (cardio), C203 (coaching), C233 (roads), C285 (city-related food, exercise), and C200 (hangouts places). The following are some examples that were composed by users with higher weight loss:

6 shirts, 2 sweaters, 6 jeans and my belts are on the last hole! I was so surprised when I read a post about new cloths and the belt was mentioned...I never knew I was already on the last hole D: I don’t need to use it, but I certainly can!

You forget that with muscles, you'll burn more fat. 80% of my training consists of strength and I do some small amount of cardio on the side.

I liked the suggestions and coaching from the jawbone app, but the overestimation is why I got a Fitbit with a heart rate monitor.

#### Diet- or Dining-Related Clusters

Diet- or dining-related clusters included C301 (nutrition), C143 (minerals), C291 (restaurant), C293 (leftover), C108 (hosting), C66 (cookout), C25 (evil gluttony), and C285 (city-related food, exercise). The following are some examples that were communicated in related posts:

today I eat a pancake with plain greek yoghurt as a pre-workout in-work snack, after gym I will inhale two greek yoghurts with two spoons of protein powder, which catapults me to 170g of protein today.

I think I've spent more time replacing bad items (like I have asparagus and Brussel sprouts instead of freezer fries now, for example) with better options than I have really giving things up.

I learned the same thing with pizza. I love pizza, but nowadays I would much rather enjoy a slice of pizza from a good local restaurant, than an entire pizza from Domino's or Papa John's.

And knowing myself before I started losing weight, if I can do it so can everyone else. I was the embodiment of laziness, gluttony and excuses.

#### Other Clusters

Other clusters included C304 (performances), C334 (negative emotions), C24 (simple, straightforward, economic), C193 (flowers), C110 (beginner), and C383 (replicate). The following are some examples of these clusters:

I ended up just drinking water at the theater because everything was either full of calories or full of sugar. ended up having some leftover chicken and a banana when I got home.

About the anxiety, yea same boat. I struggle with social anxiety since I’m 13 years old but it did get a lot better over the last few months.

Look into a Hot Pot—they are relatively inexpensive (< $15) and at least allow you to do some basics like cook rice/pasta/soup/sauces.

Finished the 13-week beginner program on DDP Yoga. Hit every workout on the schedule, didn't lag behind the schedule by even a single day once...Feels great to have stuck with it so well.

I got flowers at work! And a sweet little card that said “I'm proud of you” from my mom because of how much effort and progress I've made in the last two months.

Table 4 shows word semantic clusters that were significantly associated with lower weight loss, which are summarized below.

**Table 4:** Word semantic clusters that were statistically significant with respect to weight loss. Sample words are presented based on their distances to the center of a cluster in ascending order.

ID	Sample words	Beta	z score	SD	*P* value
C153	rising, dwindle, skyrocket, soar, rise, skyrockets, fluctuating, rises, fluctuates, spiking, spike, fluctuate	−.148	−2.782	0.053	.005
C359	purple, blue, colored, pink, brown, orange, burgundy, red, teal, gray, yellow, russet, tangerine, shades, amber	−.125	−3.464	0.036	.001
C120	request, permission, allotted, permit, requesting, requested, requests, alloted, permits, allotment, orders	−.114	−2.283	0.05	.02
C266	payments, payment, allowances, reimbursement, deduct, refund, deduction, benefits, premiums, payout	−.112	−3.282	0.034	.001
C184	vacation, vacations, getaway, vacay, resort, vacationing, resorts, honeymoon, hassle, hotels, hotel, disneyworld	−.108	−2.821	0.038	.005
C262	measurements, measurement, measuring, correlate, tracking, analysis, quantifying, monitoring, mapping	−.108	−3.627	0.030	<.001
C80	downstairs, sofa, upstairs, hallway, doorway, drawers, room, couch, cupboards, stairwell, bookshelf, fireplace	−.107	−2.022	0.053	.04
C418	hotline, contacting, contact, dialing, dialed, emailing, dial, calling, information, calls, info, referrals, referral	−.105	−5.653	0.019	<.001
C263	convince, persuade, entice, woo, coax, appease, tempt, lure, excite, inspire, convey, enlighten, quell, quench	−.101	−4.830	0.021	<.001
C421	production, producers, manufacturers, producing, output, prices, price, produce, pricing, manufacturer	−.091	−2.618	0.035	.009
C375	skinny, chubby, endomorph, lithe, pudgy, muscly, muscley, beefy, chunky, muscular, scrawny, lanky	−.084	−2.552	0.033	.01
C395	alternative, viable, workable, safe, safer, safest, alternatives, foolproof, alternate, bulletproof, inclusive	−.083	−3.455	0.024	.001
C103	tools, design, recommendations, recommended, designing, advice, recommend, materials, designs, kits	−.082	−3.075	0.027	.002
C20	split, dividing, separate, separating, splitting, divides, separated, divided, splits, divide, seperate, separation	−.070	−3.867	0.018	<.001
C227	league, scouts, rookie, leagues, teams, club, clubs, scout, seasons, season, veteran, rounder, draft	−.069	−2.911	0.024	.004
C142	budge, averse, succeeded, budging, budged, backslid, waver, incapable, qualms, bother, regressed, headway	−.062	−2.756	0.023	.006
C51	asda, morrisons, waitrose, sainsbury, sainsburys, weightwatchers, lidl, quorn, crisps, weetabix, twinings	−.060	−2.642	0.023	.008
C363	tonsils, cavities, tooth, pimples, molar, redness, toenails, teeth, sinuses, jaw, bone, zits, sinus, pimple	−.058	−2.232	0.026	.03
C18	varying, variety, varied, various, differing, different, multiple, individual, diverse, longevity, effectiveness	−.056	−3.412	0.016	.001
C124	undergraduate, college, undergrad, graduate, university, semester, graduates, semesters, academic, colleges	−.055	−2.117	0.026	.03
C92	maintenance, maintanence, maintence, maintenace, repairs, maintenence, maintainance, maintainence	−.050	−2.207	0.023	.03
C229	tweak, tweaking, tweaked, adjust, revising, updating, modifying, tweaks, modify, adjusting, readjust	−.046	−2.228	0.021	.03
C17	employed, working, work, volunteer, volunteering, volunteered, engaged, worked, engage, hire, hired	−.041	−2.127	0.019	.03
C333	moisturizer, lotion, creams, moisturizers, moisturizing, lotions, shampoo, cleanser, exfoliating, moisturising	−.034	−2.228	0.015	.03
C239	mother, daughter, aunt, niece, son, dad, grandmother, father, cousin, uncle, husband, wife, brother, sister	−.031	−2.133	0.014	.03

#### Activity-Related Clusters

Activity-related clusters included C184 (vacation), C227 (clubs, scouts), C124 (college, graduation), C92 (maintenance), and C17 (employment, volunteer). The following are some examples that were posted by users with lower weight loss:

I was fortunate enough to go on 2 European vacations this year (Italy in April, France and London 2 weeks ago) and I've gained 10lbs on top of the 15 I was already planning on losing.

At my thinnest after college I was 5'6“ 165 lbs, and after I put 20 or 30 lbs back on my mom said she was relieved because I was starting to look ”too thin.“

I'm actively seeking employment, so it depends what time I wake up if I have breakfast or not, but my usual day consists of...

My weekend was okay. Had a big unhealthy meal yesterday but stayed 200 cal. under maintenance.

#### Verb and Adjective Clusters

Verb and adjective clusters included C153 (skyrocket), C120 (request), C418 (contact hotline), C263 (entice related words), C20 (separate), C142 (backtrack), C229 (readjust), C359 (color), C375 (chunky), and C18 (various). The following are some examples:

I'm already exercising an hour 6 days a week. I really can't push it any more than that because my appetite skyrockets.

I have three diagnosed illnesses and have been hospitalized twice. Users who exhibit suicidal behavior should be pointed to suicide prevention hotlines...

I've backtracked by a couple weeks, which is partially water weight, and partially actual weight gain. It sucks.

However, I'm over my 1200 for the day, not by much but I made a silly calorie budgeting decision earlier in the day. I readjusted dinner to try to make up for it, but I was too far in the hole.

I was a chubby child, a chubby adult, and I've NEVER been in an acceptable BMI zone.

#### Expense-Related Clusters

Expense-related clusters included C226 (payment, refund), C80 (home design), C51 (supermarket), C363 (otolaryngology related issues), C421 (production, price), C333 (moisturizer), and C239 (family members). The following are some examples that were related to these clusters:

I haven't been to the gym in over a week, and honestly don't think I will unless I can come up with $350 for the remainder of my Personal Training payments...oh, and more money for actual membership dues.

I have enough motivation to get myself to the gym and am actually starting to have fun, but today I went after 4 weeks of not going, because I had my tonsils removed.

Guilty of this! I tend to drink more frequently than my husband. I was going to try to stop drinking on weekdays. However last week, for whatever reason, he had a beer 4/7 nights.

## Discussion

### Principal Findings

We used topic modeling to identify 25 general topics from the *r/loseit* subreddit. These topics covered a broad range of weight loss–related themes, including food and drinks, exercises, calorie, health issues, family members and friends, feelings, and communication. Among these topics, topics regarding food and drinks, health issues, family members and friends, calorie, and exercise were most discussed. These topics were aligned with the findings from another study [23].

Our regression analysis showed that the start weight and active days were associated with higher weight loss, which was aligned with our common sense. Furthermore, our results showed that receiving a higher karma score was associated with higher weight loss, but the median number of comments received was not significantly associated with weight loss. Our findings were a little different from the two studies, where both the karma score and comments were associated with higher weight loss [24,25]. We suspected that this might be because (1) we included far more users in our study and most of the posts received a very limited number of comments (Table 1) and (2) the previous studies did not control the model with more detailed content.

After adjusting for active days, start weight, karma score, and the median number of comments received, our analysis suggested that exercises, including coaching and nutrition, were the most effective content factors that were associated with higher weight loss, which were confirmed by previous investigations [33,34]. In addition, users with higher weight loss mentioned negative emotions that they experienced before they started to make efforts for weight loss. Our findings also suggested that mentioning food-related topics (eg, not eating too much, eating healthy food) were associated with higher weight loss, which was also found in a previous study [35]. Interestingly, we found that the mention of Xbox games was associated with higher weight loss as well. Evidence suggested that incorporating active video games had a positive effect on increasing physical activity and promoting healthy weight for both overweight adults and children [36,37].

In addition, we found that many content factors were associated with lower weight loss. For example, we found that people who mentioned vacations and clubs were more likely to have lower weight loss [38]. Furthermore, users in this subreddit mentioned that they gained weight after college graduation. Those users who had lower weight loss often mentioned supermarkets, payment or refund to exercise programs, and employment [39,40]. We also found that users who experienced health issues related to otolaryngology tended to have less weight loss. This might be due to the fact that the related treatment disturbed the weight loss plan. However, a study found that otorhinolaryngologic diseases themselves were associated with patients with obesity [41].

Another interesting finding was that users who used *skyrocket* to describe their weight loss experience (eg, feeling of eating) were less likely to have significant weight loss. This suggested that controlling the diet extensively during this process might not be an effective, healthy strategy [42]. In addition, users who often mentioned maintenance (eg, maintaining the intake of calories) were less likely to lose weight as expected. After a close examination of the related posts, we found that some of these users struggled with weight loss activities. We also found that expense-related content was associated with lower weight loss. This could be explained by a recent finding that low socioeconomic status was associated with lower weight loss outcomes [43]. Finally, users who mentioned family members were found less likely to lose more weight, suggesting that family members may not always have a positive impact on weight loss as found by other studies [44].

It should be noted that after examining feature correlation, only 6 topic predictors were included in the regression model, suggesting that word semantic clusters can capture more detailed offline activities. It was interesting that we found a calorie-related topic (T7) associated with lower weight loss, which could be partially supported by a previous finding that reducing calorie intake alone may not help in weight loss [45].

### Implications

In this study, we acknowledged that while some associations were statistically significant, the value of the coefficients was very low, indicating a weak correlation between predictors and the dependent variable. However, we did not directly interpret the predictor importance from the values of their coefficients. This is because it is practically meaningless to say that *more weight can be reduced by increasing the distribution of a certain topic discussed in an online community* [46]. Rather, we believe that it is the actual offline activities described in online discussions (or self-efficacy) that actually matter in weight loss. By using word clusters, we obtained more detailed, concrete offline activities that were often ignored by other social media–based studies but were significantly associated with the amount of weight loss. While karma scores (votes) from other users were associated with higher weight loss, considering the right-skewed distribution of karma scores (Table 1 and Multimedia Appendix 1), a majority of posts in this online community received very small karma scores.

These were somewhat aligned with the findings in an offline, SCT-based weight loss intervention program [47], where self-efficacy and intention, instead of online interaction, were found to be significant factors leading to weight loss. While self-efficacy performed well in weight loss interventions [48-50], there was evidence that self-efficacy may face the challenge of decreasing over time [51]. This is very interesting because it implied that interactions, either in online or offline environments, may serve as an indirect factor that affects weight loss through maintaining a participant’s long-term self-efficacy.

From this perspective, future weight loss analyses or interventions should consider online interaction as a key factor to improve self-efficacy, instead of directly being linked to weight loss. Our study also implied that an aggressive weight loss plan may not work in the long run.

### Limitations and Future Work

There are several limitations that we want to highlight here. First, our findings were based on merely the *r/loseit* subreddit, which constrained the generality of findings. Future work may consider extending the research to other online platforms. Second, we did not incorporate gender into the analysis. It might be possible to first infer such information from online discussions [52] and then investigate how the association between posting content and weight loss changes after controlling for this information. Third, we relied on self-reported weight loss in this study, which limited our investigation, and findings were applicable to only a small fraction of Reddit users who disclosed weight change. It will be interesting to investigate the specific characteristics that are related to the majority of Reddit users who did not report such information. Furthermore, self-reported weight changes might not be accurate because Reddit users might not update their weights in a timely manner. Our study only investigated what was presented in the online discussions, instead of examining the real-world events. Finally, it would be interesting to investigate the extent to which online interaction, not merely responses and votes but the detailed categories, and offline activities that were recorded in online discussions can lead to weight loss change in a dynamic setting.

### Conclusions

In this study, we analyzed online discussions regarding weight loss in *r/loseit*. We used topic modeling and the hierarchical clustering algorithm to extract topics and word clusters that were discussed in this subreddit. We used a regression analysis to determine the association between weight loss change and the factors that were conveyed in these online discussions. We found that the start weight and median karma scores were associated with higher weight loss. Users who had higher weight loss might be motivated by negative emotions experienced before starting weight loss. By contrast, users who mentioned vacations and payments were less likely to lose more weight. Furthermore, mentions of family members and employment were also found to be associated with lower weight loss. Our findings suggest that future interventions based on online social media platforms should focus on both online interaction and offline activities and that online personal health data can be effectively used to learn about users’ health-related behaviors.

## Appendix

Multimedia Appendix 1 Supplemental materials for selection of the optimal number of topics and the distribution of karma score.

## Abbreviations

LDA: latent Dirichlet allocation
SCT: social cognitive theory
