# Supplementary material for: Understanding Weight Loss via Online Discussions: Content Analysis of Reddit Posts Using Topic Modeling and Word Clustering Techniques
Source: J Med Internet Res. 2020 Jun 8;22(6):e13745. doi: 10.2196/13745 (PMC7308899; doi:10.2196/13745)
Supplement: Multimedia Appendix 1 [file jmir_v22i6e13745_app1.docx]

Appendix for “Understanding Weight Loss via Online Discussions: A Content Analysis of Reddit Posts Using Topic Modeling and Word Clustering Techniques”

# Yang Liu, MS^1^, Zhijun Yin, PhD^2,3^

# ^1^College of Computer Science and Technology, Changchun Normal University, Changchun City, Jilin Province, China

^2^Department of Biomedical Informatics, Vanderbilt University Medical Center, Nashville, Tennessee, USA

^3^Department of Electronical Engineering and Computer Science, Vanderbilt University, Nashville, Tennessee, USA

Corresponding Author:

Zhijun Yin, PhD

# 2525 West End Ave. 14^th^ Floor, Suite 1475, Nashville, TN, 37023,

# Department of Biomedical Informatics, Vanderbilt University Medical Center.

Email: zhijun.yin@vanderbilt.edu


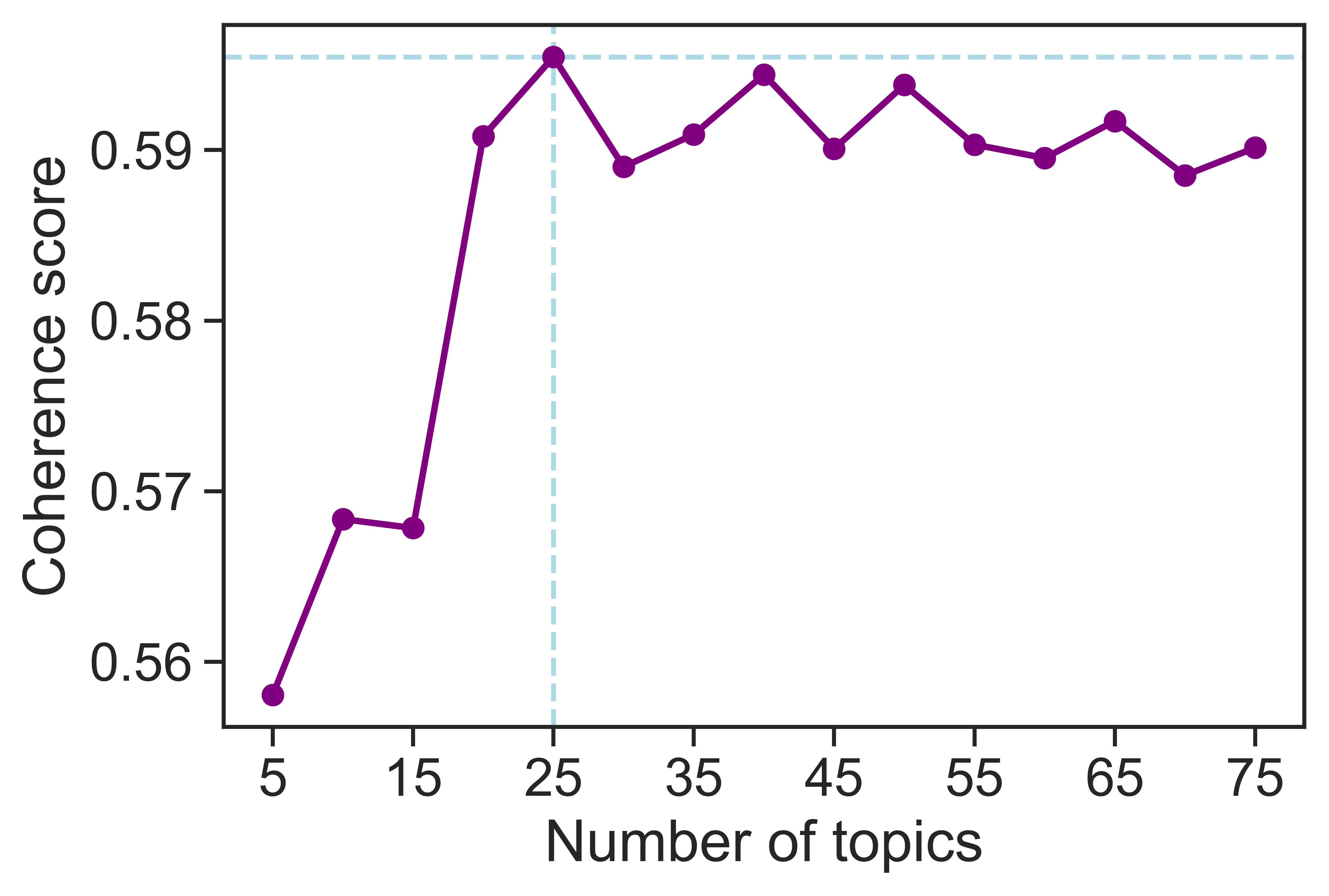


Figure A1. Illustration of coherence score with respect to the number of topics.

Figure A1 shows how the coherence score changed as the number of pre-defined topics changed. In this study, we chose 25 topics because it was corresponding to the highest score.


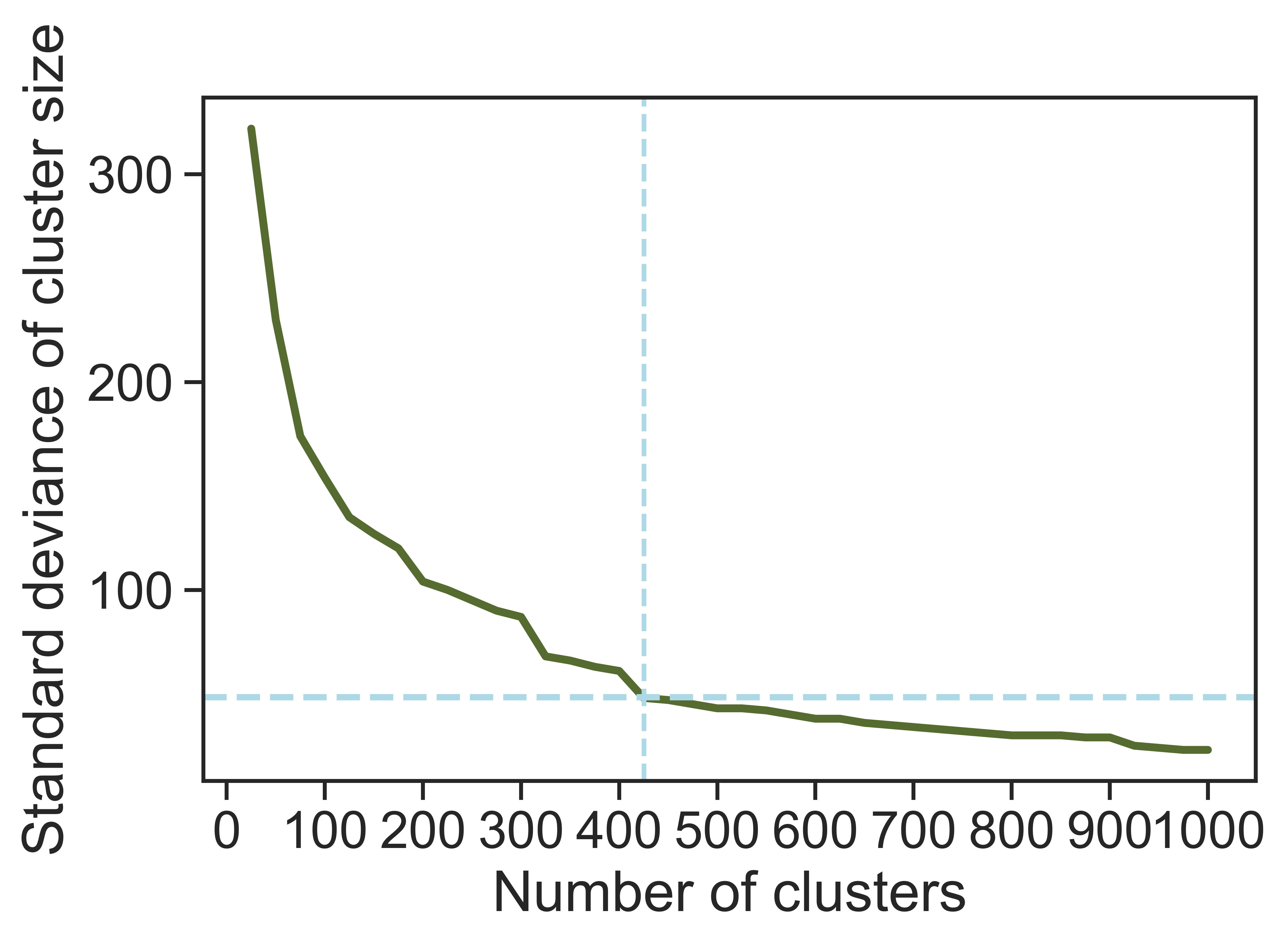


Figure A2. Illustration of standard deviation of cluster size with respect to the number of clusters.

Figure A2 shows how the standard deviation of cluster size changed over the number of clusters. Based on the elbow rule, we chose 425 word-clusters.


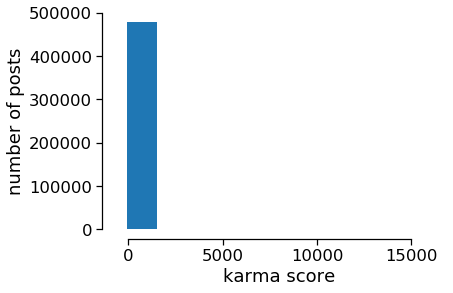


Figure A3. Histogram of karma score for each post.

Figure A3 shows the histogram of karma score for each post. From the figure, we can see that a majority of posts in r/loseit had a very limited number of karma score.
